# Nutritional and Nutraceutical Properties of Mexican Traditional Mole Sauce

**DOI:** 10.3390/molecules27030966

**Published:** 2022-01-31

**Authors:** Rafael Campos-Montiel, Gabriela Medina-Pérez, Edgar Vázquez-Nuñez, Laura Afanador-Barajas, Iridiam Hernández-Soto, Gulzar Ahmad Nayik, Lucio González-Montiel, Mohamed Alkafafy

**Affiliations:** 1 Institute of Agricultural Sciences, Autonomous University of the State of Hidalgo, Hidalgo 3600, Mexico; rgcamposm@gmail.com (R.C.-M.); iridiamsoto@gmail.com (I.H.-S.); 2 Department of Chemical, Electronic and Biomedical Engineering, Division of Sciences and Engineering, University of Guanajuato, Lomas del Bosque 103, Lomas del Campestre, León, Guanajuato 37150, Mexico; edgar.vazquez@ugto.mx; 3 Natural Sciences Department, Engineering and Sciences Faculty, Universidad Central, Bogotá 110311, Colombia; laura.afanador@gmail.com; 4 Department of Food Science & Technology, Government Degree College, Shopian 192303, India; gulzarnaik@gmail.com; 5 University of La Cañada Teotitlan de Flores Magon, Oaxaca 68540, Mexico; luciogonzalez@unca.edu.mx; 6 Department of Biotechnology, College of Science, Taif University, Taif 21944, Saudi Arabia; m.kafafy@tu.edu.sa

**Keywords:** Mexican food, mole, phenolic content, dietary fiber, mineral content, antioxidant activity

## Abstract

Mole sauce is one of the traditional Mexican foods; it is a complex mixture of ingredients of diverse origins that directly influence its nutritional value. The objective of this study was to investigate the antioxidant properties and nutritional components in five varieties of mole from Hidalgo in Mexico namely verde (V), ranchero (R), almendrado (A), casero (C), and pipian (P). Proximal chemical analysis and determination of the color index and the content of total starch, dietary fiber, mineral content (Ca, Na, K, and Mg), total phenolic content, and antioxidant activity by ABTS (2,2′-azino-bis(3-ethylbenzothiazoline-6-sulfonic acid)) and DPPH (2,2-diphenylpicrylhydrazyl) were carried out. All the five varieties of mole reported less than 25% moisture content while fat content varied from 42.9% to 58.25%. The color index ranged from a deep orange to a deep red color. A fair percentage of dietary fiber was found in all mole varieties with a low amount of starch as well. The presence of mostly insoluble dietary fiber, high phenolic content (36.13–79.49 mg GAE/100 g), and high antioxidant activity could be considered important strengths to boost the consumption of these traditional preparations. This research will contribute to a better scientific knowledge of traditional Mexican sauces as functional foods or nutraceuticals that could be used to avoid health disorders.

## 1. Introduction

Mole is a traditional Mexican food whose name comes from the Nahuatl word “*molli*” (stirred, foamy). It was handcrafted and consumed in Mexico since before the arrival of the Spaniards. In 2010, the traditional Mexican cuisine was named an “intangible cultural heritage” by UNESCO (United Nations Organization for Education, Science, and Culture) [1]. There are different kinds of mole sauces, which are classified according to their color and geographical origin; all of them comprise a complex mixture of four to seven different fresh and dry hot peppers ground with nuts, chocolate, oil, dry corn tortilla, and several spices in different proportions [2]. Mole sauces are used to prepare different kinds of dishes and marinated meats in traditional Mexican cuisine. Chili (*Capsicum annuum*) is especially important in Mexico, where it has culinary applications, such as in sauces or to give food a spicy flavor [3]; it is used in raw, boiled, dried, and ground forms [4]. Depending on the variety of mole that is made, basically three varieties of chili (*Capsicum annuum*) are used: jalapeño, guajillo, or ancho (mulato, mihuateco, and crystalline), all of them dehydrated with a moisture content of not more than 12% except green mole, i.e., verde mole, which contains fresh jalapeño pepper. The initial maturity state of the peppers influences the final color of the finished product. However, when the product is made, it is concentrated by continuous boiling, and then non-enzymatic compounds are produced due to the application of heat.

Spices and condiments are key ingredients used in the mole; to characterize its flavor, clove, cumin, pepper, cinnamon, and anise are added. They are present between the concentration of 1% to 3% in the formulations. The amount of spices added is different from one production region to another and even among different producers. It is known that some spices can also provide some other beneficial characteristics, for example having an antioxidant effect [5] or antimicrobial activity [6].

In previous research, the verde and red mole of the State of Hidalgo were physically, chemically, and microbiologically characterized, finding strengths such as protein content (11–12%), texture profile, and the absence of pathogenic microorganisms [7]. Considering that there is very little information on both the nutritional and nutraceutical value of Mexican condiments, the objective of the present study was to investigate the antioxidant capacity as well as the nutritional value (i.e., fat, protein, dietary fiber, starch, mineral content, etc.) of five traditional Mexican sauces (moles). This study will provide valuable information to increase the scientific understanding of Mexican traditional foods as functional foods or nutraceuticals, which could be useful in the prevention of chronic diseases.

## 2. Results and Discussion

### 2.1. Proximal Chemical Composition

Table 1 shows the results of the proximal chemical composition of the samples of five different varieties of mole from Hidalgo state.

In general, the protein content of the mole samples was similar, presenting ranges from 4.9% to 12.82%. Alvarez-Parrilla et al. [2] reported 2.2% to 2.5% of protein in two samples of dark mole from Guerrero state, and Guemes et al. [7] documented 2.1% to 9% of protein in moles from the State of Hidalgo. It was observed that the protein content was higher in those samples that had added seeds such as the almendrado and pipians formulations, which contain seeds of chili, pumpkin, almond, etc. The protein content of the verde mole was considerably lower, 4.68%, than reported by Guemes et al. [7] with 11.89% of protein resulting from the addition of ground pumpkin seed.

The main ingredient in a mole is fat, whose percentages were 35.47% for casero mole and 58.25% for a verde mole. Alvarez-Parrilla et al. [2] reported a fat content of 8.8% and 13.9%. The values for the crude fiber content were 3.11% fat found in the verdemole and 12.24% in the pipian mole. The fiber content is variable, which could be due to the different ingredients in different formulations as shown in the pipian in which there is a 45% addition of dry chili. There is no reference to previous work regarding fiber content. There was no siginficant difference observed among the studied moles with respect to crude fiber.

Guemes et al. [7] reported nitrogen-free extract to be in the range of 31.69% to 40.4% in the moles of the State of Hidalgo, while the results of our studied varieties were found to be between 10.70% and 38.10%. Ash content was observed to have a low concentration, with the lowest in verde (1.15%) and the highest in pipian mole (3.51%) as shown in Table 1.

### 2.2. Determination of Color Parameters

The color values L*, a *, and b * (Table 2) determine the CI (color index), which allows classification according to the color of the moles in Table 3. The results obtained show that there were no significant differences (*p* < 0.05) between the almendrado, casero, and pipian samples, although there was a significant difference (*p* < 0.05) between verde and ranchero mole.The four varieties with values within the range of +20 to +40 can be considered to be deep orange to deep red [8]. On the other hand, the verde mole with a CI of −1.082 ± 0.267 ranged from −2 to +2 and could be considered greenish yellow (Table 3).

### 2.3. Determination of Total, Soluble, and Insoluble Dietary Fiber

As shown in Table 4, there were significant differences (*p* < 0.05) between the moles in total dietary fiber (TDF); the casero, ranchero, and almendrado moles had the highest TDF among the studied moles. There were also significant differences (*p* < 0.05) among the moles in insoluble dietary fiber content (IDF), with casero, ranchero, and almendrado moles containing a higher percentage than verde and pipian moles. Regarding soluble dietary fiber (SDF), the highest content was observed in the ranchero, and the lowest in the verde mole. Significant differences (*p* < 0.05) were also observed between the samples in the case of SDF (Table 4).

### 2.4. Determination of Total Starch

Significant differences were also observed between the moles regarding the total starch content, with the highest content in the casero (3.01%) and the lowest in the pipian (0.78%) (Table 4).

### 2.5. Mineral Content

Table 5 shows significant differences (*p* < 0.05) with regard to the magnesium content, with the highest content found in the pipian mole and the lowest in the verde mole. Magnesium is an element of vital importance in the human diet with a daily requirement of 40 mg in the case of infants and up to 335 mg for pregnant or lactating mothers; it was found in concentrations ranging from 66.89 to 127.72 mg per 100 g of mole (Table 5).

No significant differences (*p* < 0.05) were found in the calcium content of the verde, pipian, and casero varieties and between the ranchero and almendrado varieties. The recommended daily intake (RDI) is 450 to 1200 mg of calcium depending on the physiological state [9]. The concentration of calcium ranged from 96.06 to 119.91 mg per 100 g of the mixture. Regarding the potassium content, significant differences (*p* < 0.05) were found between samples, with the highest content found in pipian and the lowest content in the mole verde with a concentration ranging from 552 to 1420 mg per 100 g of the mix, representing a significant contribution if one considers a recommended daily intake of 800 mg in infants to 2700 mg in adults [9]. The sodium content is directly influenced by salt (NaCl), significant differences (*p* < 0.05) were apparent in the sodium content, with the verde having the highest range and the casero mole having the lowest. Mole contains a sodium contribution of 115.77 to 315.16 mg for each 100 g. The RDI is 115 mg in infants and 3300 mg in adults. The World Health Organization advises that salt intake should not exceed 6 g per day in adults [9]. Therefore, the minimum requirement is 1 g of salt per day (400 mg of sodium). The big problem is that processed foods provide 77% of the RDI for sodium [9]. The results could be significant considering that only one formulation has added salt. The remaining four have sodium from other sources, which can justify their low sodium content and add nutritional value.

These values are for the preserved product; the consumer will have to prepare it for consumption by adding between 60% and 75% water to the preparation, which dilutes the concentrations of the original mixture.

### 2.6. Phenolic Content and Antioxidant Activity

#### 2.6.1. Total Phenolic Content

Table 6 shows the total phenolic contents of five varieties of mole; significant differences (*p* < 0.05) were found among the varieties. It was observed that the verde, which contains fresh jalapeño chili, had a lower content of phenols than the ranchero, casero, almendrado, and pipian moles, which contain mixtures of red dried chilies and have a higher range (Table 6). Ozgur et al. [10] report a phenol content for *Capsicum annuum* L. chili peppers of 55.447 ± 0.18 mg GAE/g dry weight for green chili and 89.82 ± 4.7218 mg GAE/g for red chili. They show that the content of phenolic compounds is higher in red chili peppers. These results are similar to our investigation, in which the moles with red chili peppers had a higher phenol content. Yazdizadeh Shotorbani et al. [11] also report higher phenolic content in sweet varieties (*Capsicum annuum* L.) of red chili than in green ones. Ozgur et al. [10] observed the highest range of phenolic compounds in fresh red chili peppers, reporting 130.79 ± 2.141 mg GAE/g dry weight. These results suggest that the higher content of phenols in the red moles is because the formulations of these moles contain red chili peppers.

The content of phenolic compounds found in the mole is within the ranges reported for Mexican chili peppers: 200–7820 μg of GAE/g [12,13]. In the analyzed samples, it was observed that the phenol content of the moles was lower than that reported by the authors in the pericarp of fresh chili pepper and subjected to hot air drying at different temperatures, but it must be taken into account that the mole is a complex mixture of ingredients, in which fat is added and the mixture is subjected to high heat treatment for long periods—more than 80 °C for three to four hours—conditions that can affect the content of phenolic compounds. The results regarding the total phenolic content are contradictory. Some authors report an increase in the content of phenolic compounds, while others report a decrease.

Lima et al. [14] observed a great loss of phenolic content in edible vegetables due to heat treatment. Roy et al. [15] found that lowering the temperature of treatment preserved 80–100% of the phenolic compounds in some vegetables. López et al. [16] reported that an increase in the drying temperature can significantly affect the total phenolic content in blueberries compared to that in the fresh sample. Heat treatments for drying with prolonged times at low temperatures can reduce the antioxidant effect. On the other hand, short drying times at high temperatures (90 °C) show a significant increase in the concentration of polyphenols, probably due to the generation of different antioxidant compounds with a variable degree of activity. Chen et al. [17] observed that when citrus peels were dried at 50 and 60 °C, the phenolic contents were lower than those of fresh peels; however, the phenolic content increased gradually as the drying temperature increased. The phenol content increased about twofold. Que et al. [18] indicated that the formation of phenolic compounds in pumpkin (*Cucurbita moschata* var. Duch.) occurs during drying at 70 °C and mentioned that the construction of phenolic compounds could be due to non-enzymatic interconversion between phenolic molecules.

De Jesús Ornelas-Paz et al. [4] reported that cooking operations (boiling and roasting) reduced the total phenolic content significantly (16–26.9%) in yellow, red, and green bell peppers. Likewise, Lee et al. and Chuah et al. [13,19] observed losses of phenolic compounds from non-industrial domestic cooking due to heat treatment in peppers (green, yellow, and red), squash, leeks, and other vegetables.

#### 2.6.2. Antioxidant Activity

Table 7 shows the inhibition percentages of ABTS and DPPH of the five varieties. A lower percentage of inhibition was found in the green mole (25.60%) and a higher percentage in the red ones; with values ranging from 35.30% to 41.47% inhibition of the ABTS (2,2′-azino-bis(3-ethylbenzothiazoline-6-sulfonic acid)) radical. An inhibition percentage of the DPPH (2,2-diphenyl-1-picrylhydrazyl) radical of 10.23% was observed in case of the green mole sauce and values in the range from 19.69% to 24.32% inhibition activity were displayed by red moles (Table 7). The ABTS radical inhibition values showed significant differences (*p* < 0.05). A significant difference (*p* < 0.05) was observed among the verde, casero, and pipian samples, while there was no significant difference (*p* > 0.05) found between the almond and ranch (almendrado and ranchero) varieties with respect to DPPH radical inhibition values. The ABTS and DPPH values were different because these are different methods based on different mechanisms and they do not measure the same radical. In addition, the compounds interact with each other, inducing synergistic or inhibitory effects [20,21].

The phenolic compounds found in the moles may be contributing to their antioxidant capacity, and the presence of high levels of phenols in the fruit of *Capsicum annuum* L. has been reported by some authors. Materska and Perucka [22] found high antioxidant activity of isolated fractions of flavonoids in hot pepper (*C. annuum* L.). Guil-Guerrero et al. [23] compared the antioxidant capacity of different bell pepper cultivars (*Capsicum annuum* L.) in their extracts of the chili cultivars. Matsufuji et al. [24] conducted a study on the differences in antioxidant capacity between the different colorations of the pericarp of bell pepper (*C. annuum* L.). The finding revealed that the highest radical inhibition occurred in the pericarp of the red bell pepper (around 90%) and the lowest capacity in the green one (approximately 10%). Helmja et al. [25] carried out a study in some products of the Solanaceae family (aubergine, chili, potato, and tomato) and observed an intermediate antioxidant capacity (50% inhibition of the DPPH radical) in the pungent chili (*C. annuum* L.). Ünver et al. [26] carried out study in nine spices and found that the highest antioxidant capacity measured through the inhibition of the radical DPPH was for *C. annuum* with a concentration of 0.103 mg/mL, which represents a 50% inhibition of the radical.

The results expressed in ascorbic acid equivalent milligrams for both methods (DPPH and ABTS) are shown in Table 8. Although it can be considered that the antioxidant activity is low in relation to that reported by other researchers in chili paste and in fresh chili peppers in which the temperature and drying time were controlled [21,22]. A high temperature above 80 °C, in the course of processing the mole, can modify the compounds with antioxidant activity and favor the formation of new compounds with variable antioxidant activity.

The radical scavenging activity of blueberry varieties was investigated by controlling the drying air temperature. Dehydration at high temperatures (at 80 and 90 °C) shows higher antioxidant activity rather than that at low temperatures (50, 60, and 70 °C) [16]. This behavior could be related to the low-temperature drying process, which implies long times and may cause a decrease in antioxidant activity observed.

Garau et al. [27] found the highest inhibition values of the DPPH radical scavenging effect in extracts of citrus peels (*Citrus sinensis* L.) dried at 100 °C.

## 3. Materials and Methods

### 3.1. Establishment of the Experiment

This research was carried out in the Food Science and Technology laboratories of the Food Science and Technology Research Center and the Institute of Agricultural Sciences, which belongs to the Autonomous University of the State of Hidalgo.

**Figure 1:**
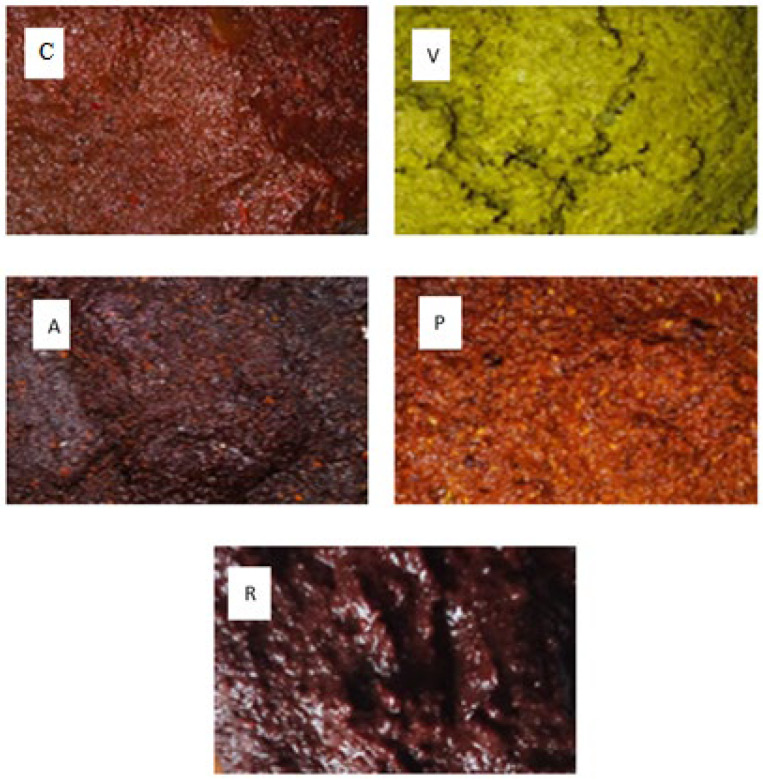
The physical state of the five mole samples from the State of Hidalgo in Mexico. They were selected according to different colorations and formulations: casero (**C**), verde (**V**), almendrado (**A**), pipian (**P**), and ranchero (**R**).

Samples of artisanal mole were collected from the State of Hidalgo; they were selected according to different colorations and formulations: ranchero (R), pipian (P), almendrado (A), casero (C), and verde (V). Figure 1 shows the physical state of the collected samples.

### 3.2. Proximal Chemical Analysis

Proximal chemical analysis of the five samples was performed using the official methods recommended by the AOAC [28]. Protein content was quantified by the Kjeldahl 950.36 method, crude fiber 950.37, ash 930.22, fat 935.38, and humidity 935.36. All tests were carried out in triplicate.

### 3.3. Color Determination

The color of the samples was determined in terms of the L *, a *, and b * values by the colorimeter (MINOLTA CM-508 d, Tokyo, Japan).

For the determination of the color index (CI), the following formula was used:

(1) $${\mathrm{CI}}^{\;}\;\frac{a\;\times 1000}{L\;\times b}\;$$

They were interpreted as follows:

(a) CI, negative (−40 to −20), its value is related to the colors ranging from blue-violet to deep green.
(b) CI, negative (−20 to −2), its value is related to the colors that go from deep green to yellowish green.
(c) CI, between (−2 to +2), its value represents greenish yellow.
(d) CI, positive (+2 to +20), is between the colors ranging from pale yellow to deep orange.
(e) CI, positive (+20 to +40), relates to deep orange to deep red colors.

### 3.4. Preparation of Samples for Dietary Fiber, Total Phenols, and Antioxidant Activity

The samples were dried by the oven method, which consisted of weighing 5 to 10 g of sample in a capsule previously set at a constant weight. The capsule was placed in the air oven for 2 h at 110 °C. After this time; it was transferred to the desiccator, where it was allowed to cool to room temperature and was weighed for later calculations. To degrease the samples, 5 g of dry sample was placed in a Büchi-brand Soxhlet extraction equipment, which consists of the ethereal extract collected in the receiving flasks, and the defatted residue from the cartridges was placed in the oven to obtain a constant weight by evaporating the solvent residues and kept in vials for later analysis.

### 3.5. Determination of Total, Soluble, and Insoluble Dietary Fiber Content

A Fiber Tech System E mod. 1023 Filtration module FOSS TECATOR was used. The amounts of total, soluble, and insoluble dietary fiber were determined by the gravimetric enzymatic method described in AOAC [28]. The enzymes used were thermostable α-amylase, amyloglucosidase, and proteases of the Sigma brand. This test was carried out using three crucibles for fiber to which 0.5 g of celite (Sigma C-8656) suspended in 10 mL of 78% alcohol was added, forming a scattered layer. They were placed in the oven at 130 °C for 12 h till a constant weight was obtained. Samples of 1 g of defatted residue were weighed in triplicate and transferred to the 500 mL flasks of the kit. Next, 50 mL of 0.05 N phosphate buffer was added at pH 6 (9.65 g of anhydrous NaH_2_PO_4_ and 1.4 g of NaH_2_PO_4_.7H_2_O were weighed, they were dissolved with 700 mL of distilled water, the pH was adjusted to 6 with 0.325 N HCl or 0.275 N NaOH and was gauged to 1 L).

The flasks were placed for 10 min in a boiling water bath under constant agitation. Without removing them, 0.1 mL of the thermostable α-amylase enzyme (Sigma A-3306) was added, and they were stirred for 15 min at the same speed. After agitation, the flasks were removed and cooled in a cold water bath to room temperature; the pH was measured and adjusted to 7.5. The flasks were placed again in the water bath, heating up to 60 °C for 10 min; without removing them, 0.1 mL of protease was added (Sigma p-3910, 25 mg of dissolved protease in 0.5 mL of phosphate buffer at pH 6), leaving them to incubate at that temperature for 30 min, keeping them under stirring. After that time, they were removed from the bath and allowed to cool to room temperature and adjusted to pH 4–4.3. They were again taken to the water bath for 10 min until they reached a temperature of 60°, later 0.1 mL of amyloglucosidase (Sigma A-9913) was added. They were incubated at the mentioned temperature for 30 min with constant shaking. At the end of that time, 95% ethanol was added while preheating to 60 °C, in an approximate ratio of ¼ (*v/v*), and they were left to rest at said temperature for 1 h. The content of each flask was vacuum filtered in each of the crucibles at a constant weight and with the celite. The residue deposited in the flasks was washed with three 20 mL portions of 78% ethanol, two doses of 95% ethanol, and two 10 mL portions of acetone. The crucibles were placed in the oven for 1.5 h at 130 °C; the weight was recorded at the end of the incineration. The sample residues from the crucibles were incinerated for 4 h at 550 °C. Two blanks were run similarly to the TDF procedure but without sample addition.

(2) $$\%\mathrm{TDF}\;=\frac{\left(\mathrm{Dry}\;\mathrm{residue}\;\mathrm{weight}-\mathrm{ash}\;-\mathrm{control}\;\mathrm{weight}\right)}{\;W}\times 100$$

where w = weight of the sample (dry base).

The percentage of IDF was determined similarly to that of TDF but without the addition of the volume of 95% alcohol. The calculations were carried out again as per those used to determine the TDF. The determination of the percentage of SDF was carried out as follows:

(3) %SDF =%TDF −%IDF

### 3.6. Determination of Total Starch

The method of Goñi and Carrón [29] was used, based on estimating the starch digestion rate through controlled hydrolysis with pancreatic α-amylase. Previously, protein hydrolysis with pepsin was carried out under conditions that emulate gastric ones. The subsequent hydrolysis of digestible starch was monitored by taking aliquots at different incubation times. The aliquots were then treated with amylo-glucosidase to achieve the total hydrolysis of the products resulting from the action of α-amylase (maltose, maltotriose, limit dextrins, etc.), determining the constituent glucose by an enzymatic-colorimetric method (GOD-PAP). The absorbance at 500 nm was read against reagent blanks; a standard curve with known amounts of glucose was plotted, and a linear regression analysis was obtained.

### 3.7. Mineral Content Quantification

Following the sample preparation method of Ferguson et al. [30] for mineral analysis, the samples were calcined to ash at 450 °C in a muffle for 12 h. After this time and when the samples were cooled, a few drops of concentrated analytical-grade nitric acid were added, and then it was returned to the muffle to recalcinate for 15–16 h. The ashes obtained were dissolved in 5 mL of analytical-grade 6N HCl and constituted to 25 mL using deionized distilled water. The Ca, Mg, Na, and K contents of triplicated solutions were determined by atomic absorption spectrophotometry (AAnalyst 400 AA).

### 3.8. Total Phenol Content and Antioxidant Activity

#### 3.8.1. Determination of Total Phenols by the Folin–Ciocalteu Method

The Folin–Ciocalteu assay has been used for many years to measure the content of total phenolic compounds in natural products. However, the basic mechanism of the method is a redox reaction, so it can be considered as another method for measuring total antioxidant activity. The method currently used is a modification made by Singleton and Rossi [31]. The oxidation of the phenols present in the sample causes the appearance of a blue coloration that has an absorption maximum at 760 nm and is quantified by spectrophotometry based on a standard gallic acid line. For the execution of this method, the Folin–Ciocalteu reagent was used at a concentration of 1 N; this reagent was protected from light and was placed under refrigeration until use. A 20% sodium carbonate (Na_2_CO_3_) solution was also prepared. The samples were prepared by placing 100 μL of the extract in glass tubes; to this, 7.9 mL of water was added and subsequently 500 μL of the Folin–Ciocalteu reagent. They were mixed in a vortex for 15 s, and then 1500 μL of the solution of 20% sodium carbonate was added and vortexed again for 15 s. It was allowed to stand for 120 min, and finally, it was read in a spectrophotometer (AAnalyst 400 AA) at 760 nm. Since the results had to be expressed in gallic acid equivalents, it was necessary to produce the respective calibration curve, where a gallic acid stock solution was prepared (20 μg/mL) previously. It was done in the dark to avoid oxidation.

#### 3.8.2. Antioxidant Activity

##### Determination of Antioxidant Capacity by Free Radical Blocking (ABTS)

The antioxidant activity was determined using the ABTS methodology developed by Re et al. [32]. The ABTS reagent was dissolved in water at a concentration of 7 mM. The ABTS radical cation was produced by reacting the ABTS stock solution with potassium persulfate (0.45 mM), and the mixture was stored at room temperature in the dark for 16–24 h before use. Subsequently, a small amount was diluted with ethanol until reaching an initial absorbance of 0.7 ± 0.20 at 734 nm (initial Abs). Briefly, 100 µL of sample in vials and 1000 µL of the reagent, diluted and stabilized at 0.7 ± 0.20 nm, were added, mixing for 30 s and immediately measuring the absorbance, and after 6 min (final Abs). The percent inhibition was calculated following Equation (4). According to the calibration curve, the results were expressed as mg of ascorbic acid per mg of ascorbic acid, with concentrations from 0 to 200 μg/mL, and their respective inhibition percentages (r^2^ = 0.990). The blanks were methanol and acetone, respectively, for the extracts of the samples. Likewise, autozero was performed with an empty cell to correct the values.

(4) $$\%\;Inhibition\;of\;ABTS=\frac{\left(Abs\;control-Abs\;sample\right)}{Abs\;control}\times 100$$

##### Determination of Antioxidant Activity by DPPH

The antioxidant activity of the samples was determined according to method adopted by Brand-Williams et al. [33], modifying some amounts, and is described below.

To prepare the 0.2 Mm DPPH solution, 7.8 mg of DPPH was dissolved in 100 mL of 80% ethanol in the dark. The preparation of the samples was done in glass tubes; 1000 μL of the 0.2 Mm DPPH solution was placed, adding 250 μL of the extract (sample), then the initial absorbance readings were taken immediately and then remained at rest and in the dark for 120 min. The readings were carried out at 517 nm in the spectrophotometer (AAnalyst 400 AA). The blank was 80% ethanol. The results were expressed as a percentage of inhibition of the radical DPPH, and the samples were analyzed in triplicate.

(5) $$\%\;Inhibition\;of\;DPPH\;radical=\frac{Abs\;control-Abs\;sample}{Abs\;control}\;\times \;100$$

where:

Abs control: is the absorbance of the blank, and

Abs test: is the absorbance of the sample.

The blanks were methanol and acetone, respectively, for the sample extracts.

### 3.9. Analysis of Results

One-way analysis of variance was applied and Tukey’s test was used to determine differences between means of the analyzed samples. All data were analyzed using the NCSS 2000, version 5, software (Wireframe Graphics, Kaysville, UT, USA). All experiments were performed in triplicate. The results were expressed as mean values ± standard deviation (SD).

## 4. Conclusions

Mole is a high-protein and high-fat food by nature of its formulation. Regarding the content of phenolic compounds in the moles, the results suggest that the higher range of phenols in the red moles is because these moles contain red chili peppers. Significant differences (*p* < 0.05) were found among the samples. It was observed that the verde mole, which contains fresh jalapeño chili, has a lower content of phenols than the other ranchero, casero, almendrado, and pipian moles, which contain mixtures of red dried chilies. The ABTS radical inhibition values showed significant differences (*p* < 0.05).

In Mexico and the United States, mole has become a popular and frequently available prepared culinary product. The outcome of this study could establish the basis for its future clinical utilization in modern food science. In addition, most of the traditional uses of mole sauce still need to be analyzed using more modern methods and further clinical trials. A few more aspects such as pharmacological, antiviral, anticancer, anti-inflammatory properties, etc., should be determined to study its phytochemical profile and health benefits.

## 5. Possible Recommendations

Prolonged heat treatment, at a temperature higher than 80 °C in the case of the mole preparation, can modify the compounds with antioxidant activity and promote the formation of new compounds with variable antioxidant activity. Moreover, the interactions of the other ingredients of the formulations, such as fat, are unknown, as fat is a complex mixture (e.g., the interaction of fat with phenolic compounds and their activity based on the processing method). Therefore, the following research stages could be used: investigation of the origin of dietary fiber, as well as its interactions with the other ingredients; correlation of the antioxidant activity of the components individually with the final antioxidant activity of the mole; evaluation of the interaction of fat with antioxidant compounds; and identification of the modified compounds with antioxidant activity formed by the interactions between carbohydrates, fats, sugars, and the application of heat.

## Figures and Tables

**Table 1:** Proximal chemical composition of five varieties of mole from the Hidalgo state in Mexico.

Mole Type	Moisture (%)	Fat (%)	Protein (%)	Crude Fiber (%)	Ash (%)	NFE (%)
V	21.74 ± 1.08 ^c^	58.25 ± 0.43 ^d^	4.90 ± 0.25 ^a^	3.20 ± 0.11 ^a^	1.15 ± 0.05 ^a^	10.7 ± 0.43 ^a^
R	13.43 ± 0.60 ^b^	39.33 ± 0.03 ^b^	8.53 ± 0.42 ^b^	3.80 ± 0.09 ^a^	2.38 ± 0.10 ^b^	16.97 ± 0.45 ^b^
C	11.30 ± 0.67 ^a^	35.47 ± 0.13 ^a^	9.53 ± 0.57 ^bc^	3.50 ± 0.17 ^a^	3.04 ± 0.09 ^c^	31.56 ± 1.01 ^c^
A	13.26 ± 0.92 ^b^	36.65 ± 0.24 ^a^	10.78 ± 0.75 ^c^	3.10 ± 0.09 ^a^	3.13 ± 0.47 ^c^	33.06 ± 0.08 ^c^
P	11.50 ± 0.43 ^a^	42.90 ± 0.19 ^c^	12.82 ± 0.64 ^d^	12.20 ± 0.98 ^b^	3.51 ± 0.54 ^c^	38.10 ± 0.33 ^d^

The superscripts a, b, c, and d show significant differences (*p* < 0.05) among the moles using the Tukey mean comparison technique. NFE: nitrogen-free extract. verde (V), ranchero (R), casero (C), almendrado (A) and pipian (P).

**Table 2:** Values of L*, a*, and b* color five varieties of mole from the State of Hidalgo.

Mole Type	L*	b*	a*
V	45.49 ± 0.10 ^c^	16.79 ± 0.13 ^c^	0.82 ± 0.38 ^d^
R	27.34 ± 0.50 ^a^	5.80 ± 0.51 ^a^	3.51 ± 0.06 ^a^
C	30.43 ± 0.48 ^b^	7.79 ± 0.24 ^b^	6.92 ± 0.26 ^b^
A	31.87 ± 0.58 ^b^	8.47 ± 0.31 ^b^	8.00 ± 0.41 ^c^
P	28.37 ± 0.45 ^a^	8.01 ± 0.30 ^b^	6.72 ± 0.17 ^b^

The superscripts a, b, c, and d show significant differences (*p* < 0.05) among the moles using the Tukey mean comparison technique; verde (V), ranchero (R), casero (C), almendrado (A) and pipian (P).

**Table 3:** The color index of five varieties of mole from the State of Hidalgo.

Mole Type	Color Index (CI)	Interval
V	−1.08 ± 0.26 ^c^	Greenish yellow (−2 to +2)
R	22.22 ± 0.20 ^b^	Deep orange to deep red ( +20 to +40)
C	29.17 ± 0.17 ^a^	Deep orange to deep red (+20 to +40)
A	29.63 ± 0.10 ^a^	Deep orange to deep red (+20 to +40)
P	29.580 ± 0.26 ^a^	Deep orange to deep red (+20 to +40)

The superscripts a, b and c show significant differences (*p* < 0.05) among the moles using the Tukey mean comparison technique; verde (V), ranchero (R), casero (C), almendrado (A) and pipian (P).

**Table 4:** The total soluble and insoluble dietary fiber content of five varieties of mole from the State of Hidalgo.

Mole Type	TDF%	IDF%	SDF%	Total Starch%
V	4.60 ± 0.09 ^a^	4.30 ± 0.01 ^a^	0.29 ± 0.01 ^a^	1.08 ± 0.02 ^b^
R	12.20 ± 0.08 ^c^	10.40 ± 0.01 ^c^	1.89 ± 0.08 ^c^	2.42 ± 0.05 ^c^
C	11.50 ± 0.02 ^c^	10.20 ± 0.02 ^c^	1.28 ± 0.04 ^b^	3.01 ± 0.01 ^d^
A	11.80 ± 0.02 ^c^	10.03 ± 0.02 ^c^	1.46 ± 0.01 ^b^	2.36 ± 0.04 ^c^
P	9.80 ± 0.01 ^b^	8.30 ± 0.01 ^b^	1.53 ± 0.02 ^b^	0.78 ± 0.01 ^a^

TDF = total dietary fiber, IDF = insoluble dietary fiber, SDF = soluble dietary fiber. The superscripts a, b, c, and d show significant differences (*p* < 0.05) among the moles using the Tukey mean comparison technique; verde (V), ranchero (R), casero (C), almendrado (A) and pipian (P).

**Table 5:** Mineral content in five mole types from Hidalgo state, Mexico.

Mole Type	Magnesium	Sodium	Potassium	Calcium
	Mg	Na	K	Ca
	mg/100 g	mg/100 g	mg/100 g	mg/100 g
V	66.89 ± 2.36 ^a^	315.16 ± 36.50 ^c^	552.85 ± 3.21 ^a^	110.91 ± 0.21 ^a^
R	91.25 ± 1.78 ^b^	127.93 ± 10.47 ^a^	999.83 ± 10.08 ^b^	96.47 ± 2.74 ^b^
C	105.31 ± 0.94 ^c^	115.77 ± 3.09 ^a^	1279.58 ± 4.38 ^d^	119.91 ± 5.03 ^a^
A	111.33 ± 0.94 ^c^	181.93 ± 4.66 ^b^	987.79 ± 18.63 ^b^	96.06 ± 1.37 ^b^
P	127.72 ± 3.22 ^d^	125.22 ± 4.96 ^a^	1420.5 ± 40.40 ^c^	115.08 ± 6.24 ^a^

The superscripts a, b, c, and d show significant differences (*p* < 0.05) among the moles using the Tukey mean comparison technique; verde (V), ranchero (R), casero (C), almendrado (A) and pipian (P).

**Table 6:** Total phenolic content in five mole types from Hidalgo state, Mexico.

Mole Type	mg GAE/100 g (dw)
V	36.13 ± 0.27 ^a^
R	49.13 ± 0.48 ^b^
C	50.23 ± 0.53 ^b^
A	79.49 ± 0.35 ^d^
P	56.03 ± 0.40 ^c^

The superscripts a, b, c, and d show significant differences (*p* < 0.05) among the moles using the Tukey mean comparison test. GAE—gallic acid equivalent; verde (V), ranchero (R), casero (C), almendrado (A) and pipian (P).

**Table 7:** Percentages of inhibition of five varieties of moles of the State of Hidalgo by the method of ABTS and DPPH.

Mole Type	ABTS%	DPPH%
V	25.60 ± 0.67 ^a^	10.23 ± 0.22 ^a^
R	37.48 ± 0.48 ^b^	24.32 ± 0.45 ^d^
C	41.47 ± 0.86 ^d^	19.69 ± 0.58 ^b^
A	39.95 ± 0.44 ^c^	23.58 ± 0.075 ^d^
P	35.30 ± 0.92 ^b^	21.51 ± 0.42 ^c^

The superscripts a, b, c, and d show significant differences (*p* < 0.05) among the moles using the Tukey mean comparison test. ABTS: (2,2′-azino-bis(3- ethylbenzothiazoline-6-sulfonic acid), DPPH: (2,2-diphenyl-1-picrylhydrazyl). verde (V), ranchero (R), casero (C), almendrado (A) and pipian (P).

**Table 8:** Antioxidant activity of five types of mole from the State of Hidalgo, by the ABTS and DPPH method expressed in ascorbic acid equivalents (mg AAE/100 g of dry base sample).

Mole Type	ABTS mg AAE/100 g	DPPH mg AAE/100 g
V	77.67 ± 0.47 ^a^	87.38 ± 0.82 ^a^
R	92.12 ± 0.94 ^c^	116.32 ± 0.58 ^d^
C	96.97 ± 1.19 ^d^	106.82 ± 1.05 ^b^
A	95.12 ± 0.16 ^d^	114.82 ± 0.55 ^d^
P	89.47 ± 0.88 ^b^	110.56 ± 1.13 ^c^

The superscripts a, b, c, and d show significant differences (*p* < 0.05) among the moles using the Tukey mean comparison test; ABTS: (2,2′-azino-bis(3-ethylbenzothiazoline-6-sulfonic acid), DPPH: (2,2-diphenyl-1-picrylhydrazyl, AAE: ascorbic acid equivalents. verde (V), ranchero (R), casero (C), almendrado (A) and pipian (P).

## Sample Availability

Samples are available from the authors.
